# Predictive Parameters of Arteriovenous Fistula Functional Maturation in a Population of Patients with End-Stage Renal Disease

**DOI:** 10.1371/journal.pone.0119958

**Published:** 2015-03-13

**Authors:** Khalid Bashar, Adeel Zafar, Sawsan Elsheikh, Donagh A. Healy, Mary Clarke-Moloney, Liam Casserly, Paul E. Burke, Eamon G. Kavanagh, Stewart R. Walsh

**Affiliations:** 1 Department of Vascular Surgery, University Hospital Limerick, Limerick, Ireland; 2 Department of Acute Medicine, James Connolly Memorial Hospital, Dublin, Ireland; 3 Department of Nephrology, University Hospital Limerick, Limerick, Ireland; 4 Department of Surgery, National University of Ireland, Galway, Ireland; University of Florida, UNITED STATES

## Abstract

**Introduction:**

With increasing numbers of patients diagnosed with ESRD, arteriovenous fistula (AVF) maturation has become a major factor in improving both dialysis related outcomes and quality of life of those patients. Compared to other types of access it has been established that a functional AVF access is the least likely to be associated with thrombosis, infection, hospital admissions, secondary interventions to maintain patency and death.

**Aim:**

Study of demographic factors implicated in the functional maturation of arteriovenous fistulas. Also, to explore any possible association between preoperative haematological investigations and functional maturation.

**Methods:**

We performed a retrospective chart review of all patients with ESRD who were referred to the vascular service in the University Hospital of Limerick for creation of vascular access for HD. We included patients with primary AVFs; and excluded those who underwent secondary procedures.

**Results:**

Overall AVF functional maturation rate in our study was 53.7% (52/97). Female gender showed significant association with nonmaturation (P = 0.004) and was the only predictor for non-maturation in a logistic regression model (P = 0.011). Patients who had history of renal transplant (P = 0.036), had relatively lower haemoglobin levels (P = 0.01) and were on calcium channel blockers (P = 0.001) showed better functional maturation rates.

**Conclusion:**

Female gender was found to be associated with functional non-maturation, while a history kidney transplant, calcium channel-blocker agents and low haemoglobin levels were all associated with successful functional maturation. In view of the conflicting evidence in the literature, large prospective multi-centre registry-based studies with well-defined outcomes are needed.

## Introduction

The number of patients with end stage renal disease (ESRD) has been increasing steadily, a trend which is expected to continue; as a result, more patients are expected to require vascular access placement for haemodialysis (HD) [1,2]. A mature and functional arteriovenous fistula (AVF) is considered the best modality for HD access when compared to arteriovenous grafts (AVG) and central venous catheters (CVC) [3–5], however it is expected that approximately one third (20%–50%) of AVFs will fail to mature into useful access [6–8]. Although the chances for an AVF to fail are high, they should still be considered first in all patients planned to start HD sessions, and for the ones who have already started HD. Mortality rate has been shown to be significantly higher in those who dialyse first by means of tunnelled catheters, and at the same time, they are at increased risk of failure of subsequent AVF [7,9,10]. Arteriovenous grafts tend to have better primary patency rates compared to AVF [6,11,12], however AVFs last longer, and with the exception of those fistulas which fail to mature primarily, the cumulative patency (from formation to permanent failure) is superior to grafts; moreover, AVFs—once they fully mature—are less likely to require secondary procedures for vascular access salvage to maintain patency, including angioplasty, stenting or thrombectomy [6,13–16]. The 2006 updated NKF-KDOQI Guidelines recommend AVF prevalence of ≥ 65% for patients undergoing HD [17]. Currently, the prevalence of AVF in those patients is around 80% in Europe and around 60% in the United States [7,10,18].

Certain patients’ characteristics have been associated historically with poor AVF maturation rates, in particular female gender, age and diabetes. Conte MS et al published study of 31 patients who had AVFs created as part of their V-HEALTH trial. They found that diabetic patients had significantly lower patency rates in the 24 weeks of the follow-up period [19]. Similarly, Salmela et al reported that diabetes, female sex and thrombophilia were all associated with decreased primary fistula patency rates [20]. Conversely, Sedlacek et al in study of 195 patients reported that diabetes was not associated with AVF maturation (67% matured in the diabetic group vs. 62% in non-diabetic group); also diabetes did not influence the prevalence of AVF creation as 66% in diabetic group underwent fistula placement compared to 60% in the non-diabetic group [21]. More recently Allon et al found that both age and diabetes were not associated with increased non-maturation rates, although they were both significantly linked to increased medial fibrosis [22].

Another factor thought to be associated with AVF maturation is age. Elderly patients are traditionally thought to have worse patency rates and more likely to suffer from AVF non-maturation [23,24]. However, this has been disputed by other authors [8,25].

With regards to the association between gender variation, and AVF non-maturation, there have been conflicting results reported in published literature. Several studies suggested a significant correlation between female gender and decreased patency rates in AVFs, as well as prolonged maturation time before the fistula can be used adequately to sustain HD sessions [20,23,26]. A combination of female gender and increased age (> 65) has been shown to be significantly associated with non-maturation when compared to men of the same age group [22,24]. However, several other studies found no significant association between female gender and high risk of AVF non-maturation [22,25,27].

Certain haematological findings have been implicated in the maturation process of AVF. Khavanin Zadeh et al in a prospective study of HD patients who were referred for first time AVF formation reported higher risk of AVF failure in those with haemoglobin level < 8 g/dl (RR = 1.41; p = 0.01) [28]. More recently, Yilmaz et al looked into the relationship between late AVF stenosis and neutrophil-lymphocyte-ratio (NLR) based on blood results obtained from chronic haemodialysis patients. They hypothesised that increased level of inflammatory markers will lead to increased number of AVF stenosis cases. They suggested that the mechanisms responsible for AVF stenosis might be similar to those involved in atherosclerosis disease [29].

The objective of this paper was to report our own findings from the last 7 years in a regional hospital situated in the Mid-Western area of the Ireland in relation to patients’ characteristics and comorbidities that might affect the process of AVF maturation according to predefined outcomes. We aimed to test the hypothesis that certain patients’ characteristics (age, gender and medical co-morbidities—diabetes in particular) affect the maturation of AVF. We also aimed to test the association between specific inflammatory markers (white cell count and neutrophils) and haemoglobin’s level preoperatively with AVF maturation.

## Methods

### Patients

We performed a retrospective chart review of all patients with ESRD who were referred to the vascular service in the University Hospital of Limerick for creation of vascular access for HD. Three surgeons performed the procedures. The data-analysis was performed according to a predefined set of outcomes based on extensive search of the literature.

### Inclusion and exclusion criteria

We included all patients aged 18 years or older who underwent formation of AVF in the upper limb between 2006 and 2013. Patients with multiple episodes, each episode was considered separately and data from the corresponding episode was recorded on our data sheet. We excluded patients that underwent salvage procedures to improve maturation, i.e. secondary maturation, as we analysed data related to primary functional maturation rates only. All patients who had prosthetic graft and/or tunnelled catheters as the only means for HD were excluded.

### Data collection

After obtaining an ethical approval for the study from the research ethics committee and the risk management department of the regional Health Service Executive (HSE West), data for all included patients were extracted from their medical records. Patients were not asked to provide consents (written/oral), as all records were anonymised and data were de-identified prior to analysis and reporting of findings. Baseline demographic information, site and type of the AV fistula were retrieved from the medical records, whereas results of blood investigations were obtained from electronic records. Functional maturation was recorded from dialysis records.

### Study’s primary and secondary endpoints

We aimed to evaluate patients’ characteristics that have been reported to be associated with AVF non-maturation in the literature following an extensive review of published evidence. We used functional maturation in this study which was defined as successful use of the arteriovenous fistula for 6 consecutive sessions of HD, and this was obtained from dialysis records. The use of functional maturation defined as sustained HD sessions ≥ 6 for the evaluation of AVF maturation has been validated in the literature in several previous studies [30–32]. While this method is acceptable, particularly in retrospective studies assessing AVF maturation before the regular use of preoperative venous mapping and postoperative US scans—as the case with this study as most of our patients did not have postoperative US scans—we should however point to its inferiority compared to the definition recommended in the updated NKF-KDOQI guidelines, famously known as the rule of (6s) (flow of approximately 600 mL/min, less than 0.6 cm below the skin surface and a minimal diameter of 0.6 cm) [17]. Also, in the absence of postoperative imaging scans, it will be difficult if not entirely impossible to differentiate between non-maturation and mis-cannulation due to less experienced staff. At our hospital, new fistulas are looked after by senior dialysis nurses who are well experienced in cannulation of those fistulas, however, it should be emphasised that the US based definition for AVF maturation adopted by the NKF-DOQI is superior to the one we used.

We examined the relationship between age, gender, diabetes, smoking, hypertension, hyperlipidaemia, history of steroids use, history of Calcium channel blockers at the time of the access formation and the history of previous dialysis access (AVF, AVG or CVC). Secondary endpoints were perioperative blood investigations (Haemoglobin, White cells count and Neutrophils count). We also recorded the aetiology behind ESRD whenever available from medical records.

### Statistical analysis

Data were extracted and recorded on spread sheet using IBM SPSS version 22.0 [33]. Categorical data are expressed in true value and as percentages and were compared using the Pearson Chi-Square (X^2^) test, whereas continuous data were reported as mean ± SD and compared using the independent sample t-test for normally distributed data, and the Mann-Whitney U when indicated by normality tests. Levene’s test for equality of variances was used to determine the p value in t-test regression analysis for continuous data [34]. Data distribution of various predictor variables were assessed by means of histograms, Q-Q plots and box plots. Finally, a prediction model was calculated by logistic regression analysis using data from variables that have been suggested to correlate to fistula in the literature, as well as variables from our study with a *p* value of < 0.1 in bivariate analysis with functional maturation being the dependent (outcome) measure of analysis. We also performed an overall logistic regression test with all of the included variables in our study without restrictions in terms of the *p* value.

## Results

**Fig 1:**
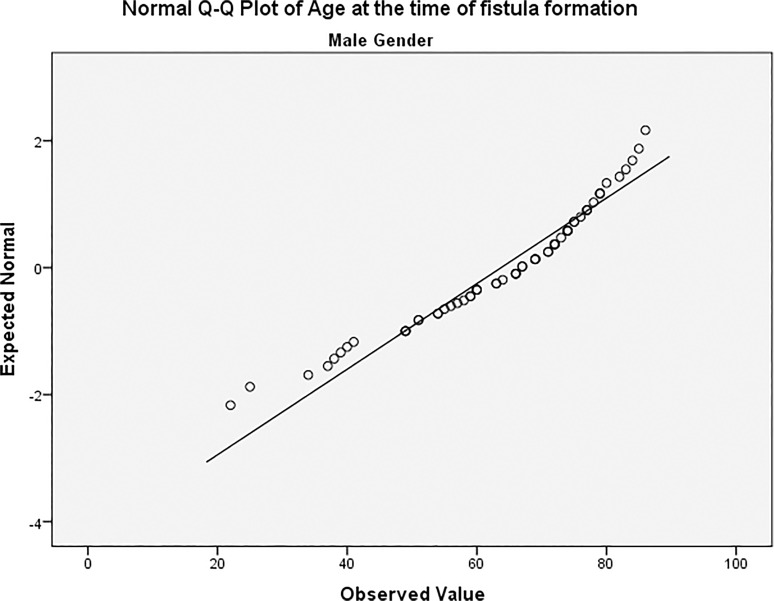
Age distribution among male patients.

**Fig 2:**
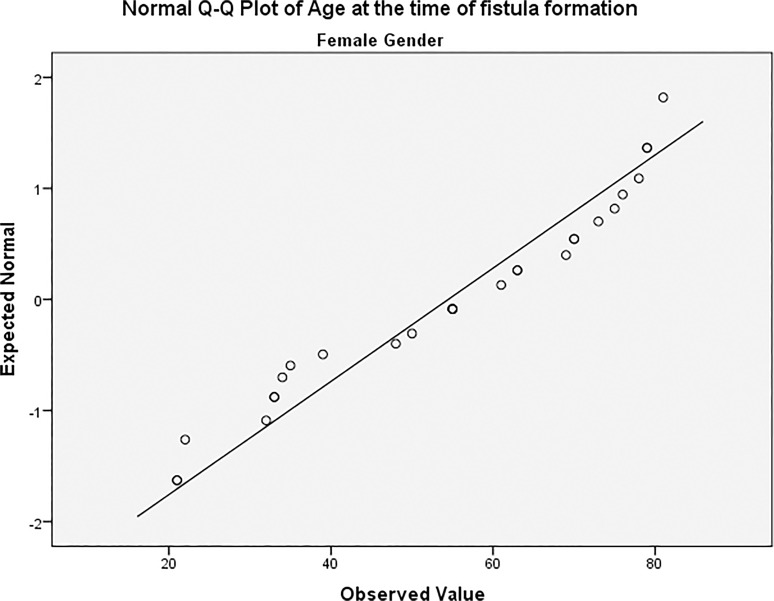
Age distribution among female patients.

**Fig 3:**
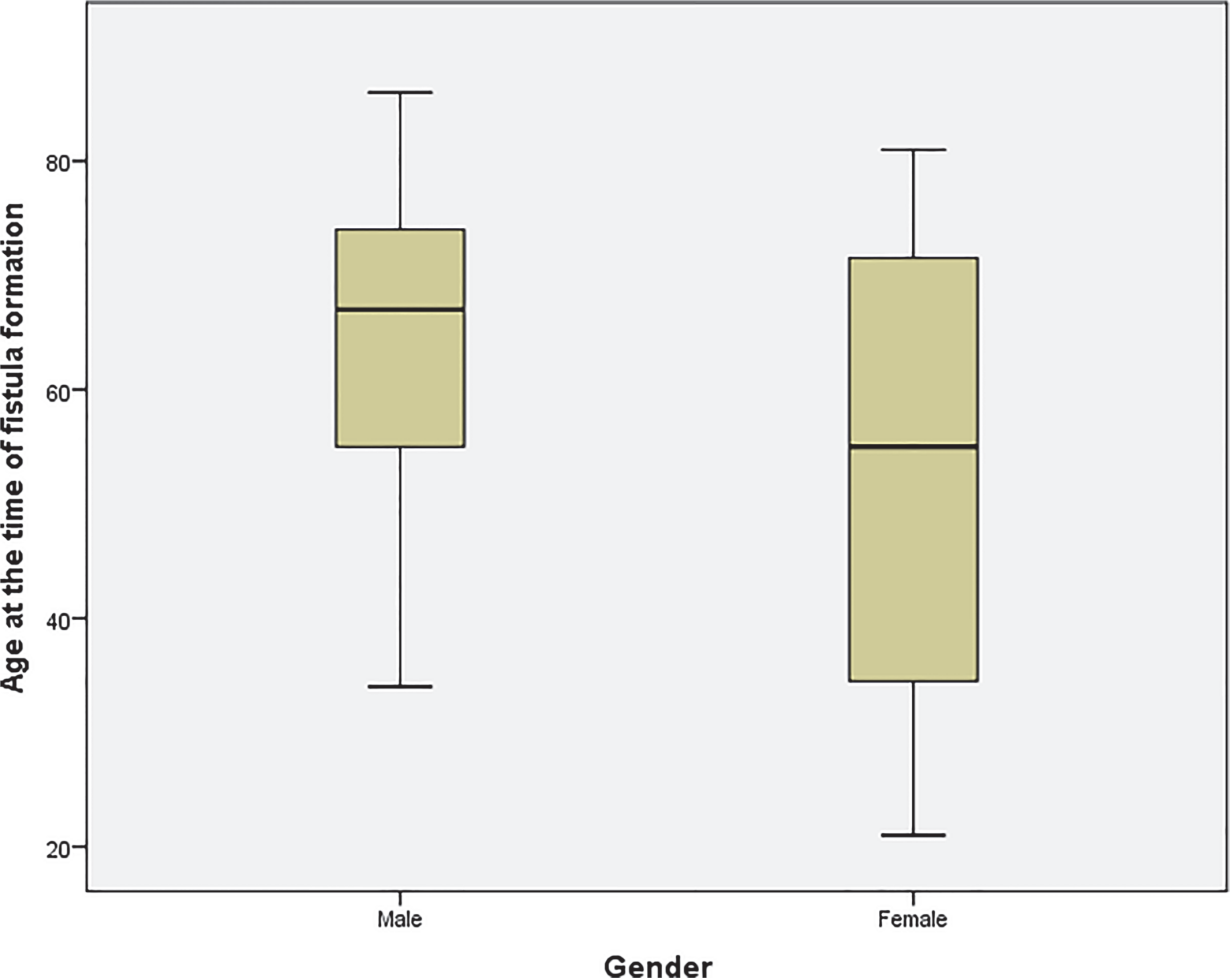
Variation in age between males and females groups.

The study included a total of 86 patients (all diagnosed with ESRD by their attending consultant nephrologists and referred to the vascular department for access creation) with 97 arteriovenous fistulas formed to serve as vascular access for HD sessions. The most common cause leading to ESRD was diabetes (n = 37/97; 38.1%) followed by congenital renal agenesis (8/97; 8.2%), hypertension (7/97; 7.2%) and ischaemic injury (6/97; 6.2%). Other diagnosis included vasculitis, hypercalcaemia and a number of autoimmune diseases. From the 97 AVFs included in the study, 68 (70.1%) were constructed in men while 29 (29.9%) were constructed in female patients. Age did not follow a normal distribution with regards to gender variation in our patients [Figs. 1 and 2]. Age of all included patients was (mean ± SD) 60.9 ± 16.9; men aged 63.7 ± 14.8 with a median of 67 (22–86) while women aged 54.5 ± 19.6 with a median of 55 (21–81); this difference was statistically significant (P = 0.012) [Fig. 3]. Demographic data of included patients along with comorbidities and drug therapy at the time of fistula formation are summarised in [Table 1], and summary of the continuous variables in our study can be found in [Table 2].

**Table 1:** Characteristics of patients in study.

	Frequency and percentage of the positive (yes) values	
Baseline characteristic	Frequency (Observed %)	Valid %, N = 97
Functional maturation	52 (53.6)	57.6
Female gender	29(29.9)	29.9
Male gender	68 (70.1)	70.1
Diabetes	40 (41.2)	41.2
Smoking	30 (30.9)	32.3
Hypertension	81 (83.5)	85.3
Hyperlipidaemia	71 (73.2)	74.7
Coronary artery disease	35 (36.1)	36.8
Atrial fibrillation	15 (15.5)	15.8
Warfarin	16 (15.5)	15.8
Congestive cardiac failure	24 (24.7)	25.3
Insulin	17 (17.5)	18.1
Calcium channel blockers	30 (30.9)	32.3
Previous history of haemodialysis	69 (71.4)	73.4
Dialysis through Venous Catheter	64 (66)	70.3
Previous kidney transplant	9 (9.3)	10.1
Previous Arteriovenous fistula	21 (21.6)	32.8
Site of AVF: Wrist	45 (46.4)	50
Site of AVF: Forearm	45 (46.4)	50

AVF = arteriovenous fistula.

**Table 2:** Characteristics of continuous variables.

	Age at creation of fistula	Urea (mg/dL)	Creatinine (μmol/L)	Haemoglobin (g/dl)	Platelets (10^9^/L)	White Cells Count (10^9^/L)	Neutrophils Count (10^9^/L)
Mean	60.92	17.915	528.42	11.049	252.73	7.6639	5.3045
Std. Deviation	16.858	8.8747	260.563	1.6074	111.809	2.08253	1.82589
Median	66.00	16.800	448.00	11.000	234.00	7.2900	4.9400
Minimum	21	1.8	214	7.2	110	3.58	2.20
Maximum	86	45.4	1506	14.5	1007	14.04	12.11

### Patients’ characteristics

Overall AVF functional maturation rate in our study was 53.6% (52/97). If we excluded the 9 patients that did not have sufficient information to confidently establish maturation according to the definition used from their medical records, the functional maturation rate was 57.6%.

**Fig 4:**
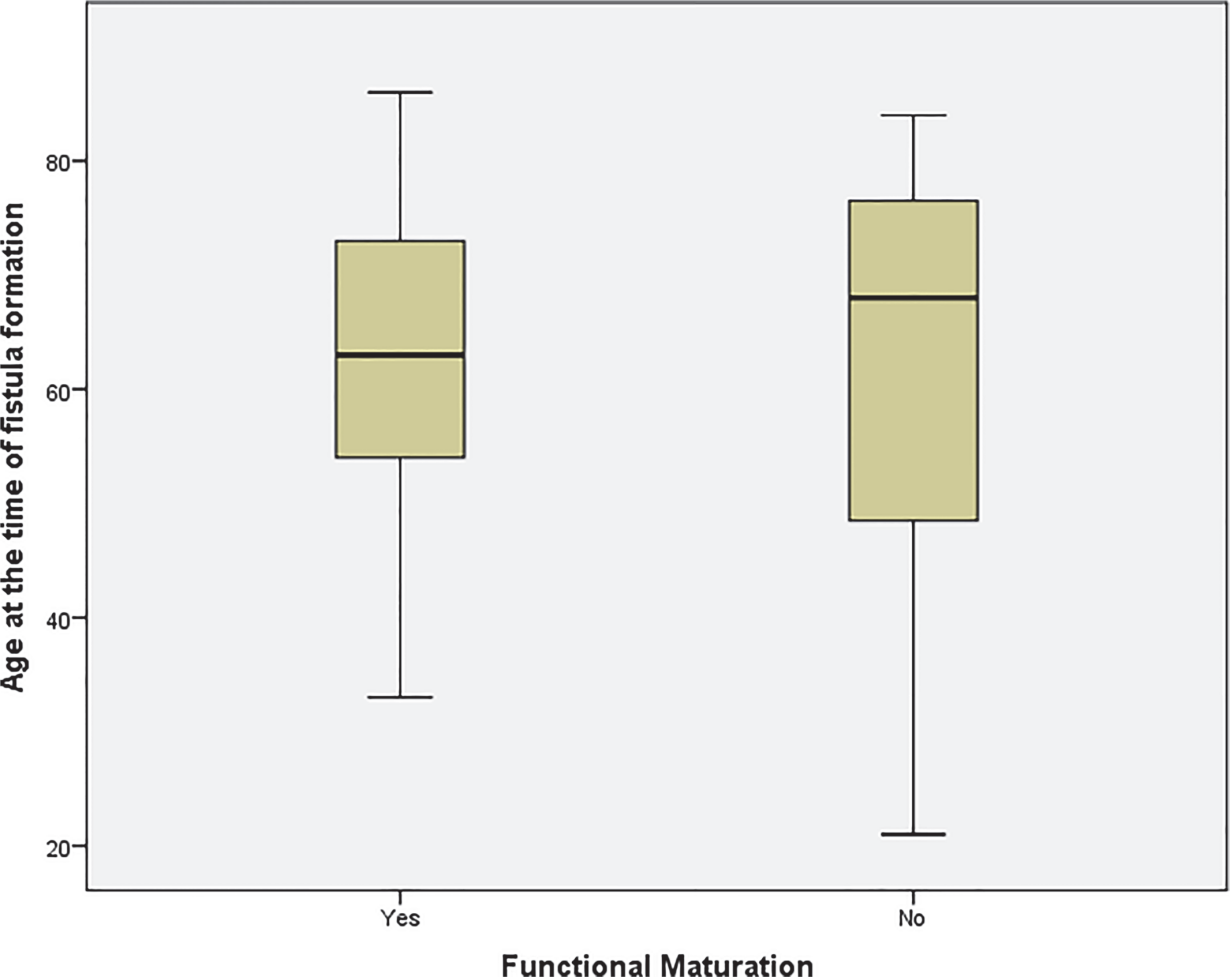
Variation in age between mature and non-mature AVF groups.

We examined the relationship between different patients’ characteristics, comorbidities and drug therapy and functional maturation in our patients using the appropriate statistical tests as outlined above; 40/59 (67.8%) fistulas matured in men while 9/26 (34.6%) matured in female patients; this difference was statistically significant (P = 0.004) suggesting female gender is associated with poor functional maturation. Age was not distributed equally among patients with a documented maturation outcome; however it was not found to be statistically associated with functional maturation as those who were found to have a functional access aged (62.4 ± 13.9) compared to (60 ± 20) in patients who could not dialyse from their AVFs (P = 0.926; Mann-Whitney U test) [Fig. 4]. Hypertension was diagnosed in 72 cases; 44 (61.1%) of those matured, whereas of the 13 cases who did not suffer from hypertension five (38.5%) fistulas matured (P = 0.128). Of the 34 fistulas with a positive diagnosis of diabetes 18 fistulas of those matured whereas 16 did not mature, compared to 31 and 20 respectively of the 51 patients who had a documented different aetiology for ESRD. The difference between the two groups of patients was not significant (P = 0.473). The use of Insulin for the treatment of diabetes also did not correlate significantly to functional maturation (P = 0.839).

Being on a calcium channel blocker at the time of fistula formation was significantly associated with a more favourable outcome as 21/25 patients on those medications had mature fistulas compared to 27/59 of patients who were not on calcium channel blockers (P = 0.001). There was no statistically significant difference in functional maturation if the patient was on Aspirin and/or Clopidogrel, or neither of the two drugs (P = 0.617). Previous history of HD was not found to be statistically related to functional maturation, as 37/60 functional AVFs were created successfully in patients with previous access (AVF, AVG or CVC) whereas 12/25 non-mature fistulas were created in patients with previous access (P = 0.245), even when we performed separate analysis on those who only dialysed via tunnelled catheters, i.e. excluding AVF and AVG, the result remained insignificant (P = 0.407). Also, those who underwent a new fistula formation for a failed previous AVF did not do any worse or better in terms of functional maturation (P = 0.530). However, history of a previous kidney transplant surgery was found to be associated with better functional maturation (P = 0.036).

The site of the newly created AVF was not found to statistically influence the outcome of functional maturation in our study; of the 43 fistulas created around the wrist, 23 (53.5%) matured and 20 (46.5%) failed, compared to 26 (61.9%) and 18 (38.1%) of those placed in the forearm respectively (P = 0.432). The association between functional maturation and other categorical variables are shown in [Table 3].

**Table 3:** Categorical variables association with functional maturation.

Variable	Functional maturation	N	%	P value
Female gender	Yes	9	34.6	**0.004**
	No	17	65.4	
Male gender	Yes	40	67.8	
	No	19	32.2	
Diabetes	Yes	18	52.9	0.473
	No	16	47.1	
Smoking	Yes	13	48.7	0.193
	No	14	51.9	
Hypertension	Yes	44	61.1	0.128
	No	28	38.9	
Hyperlipidaemia	Yes	38	57.6	0.980
	No	28	42.4	
CAD	Yes	17	54.8	0.691
	No	14	45.2	
Atrial fibrillation	Yes	8	66.7	0.495
	No	4	33.3	
PVD	Yes	10	58.8	0.913
	No	7	41.2	
HD history	Yes	37	61.7	0.245
	No	23	38.3	
HD by central catheter	Yes	35	61.4	0.407
	No	22	38.6	
Previous AVF	Yes	11	55	0.530
	No	9	45	
Site of AVF: Wrist	Yes	23	61.9	0.432
	No	20	38.1	
Site of AVF: Forearm	Yes	26	65.4	
	No	16	34.6	
Renal transplant history	Yes	8	88.9	**0.036**
	No	1	11.1	
C^+2^ channel blocker	Yes	21	84	**0.001**
	No	4	16	
Warfarin	Yes	8	61.5	0.758
	No	5	38.5	
Insulin	Yes	9	60	0.839
	No	6	40	

CAD = coronary artery disease; PVD = peripheral vascular disease; HD = haemodialysis, AVF = arteriovenous fistula; AVF = arteriovenous fistula.

### Preoperative blood tests

We also examined the relationship between functional maturation and a number of blood based investigations obtained within 24 hours prior to the time of access placement, we used (mean ± SD) to report and compare our findings. Fistulas which were used successfully for HD had a haemoglobin level of (10.6 ± 1.5 g/dl), platelets count (252.2 ± 136.1 10^9^/L), white cells count (7.6 ± 2.2 10^9^/L) and neutrophils count of (5.4 ± 1.9 10^9^/L) compared to (11.5 ± 1.7 g/dl), (263 ± 82.4 10^9^/L), (8 ± 2 10^9^/L) and (5.5 ± 1.8 10^9^/L) in patients with non-mature fistulas respectively [Table 2].

**Fig 5:**
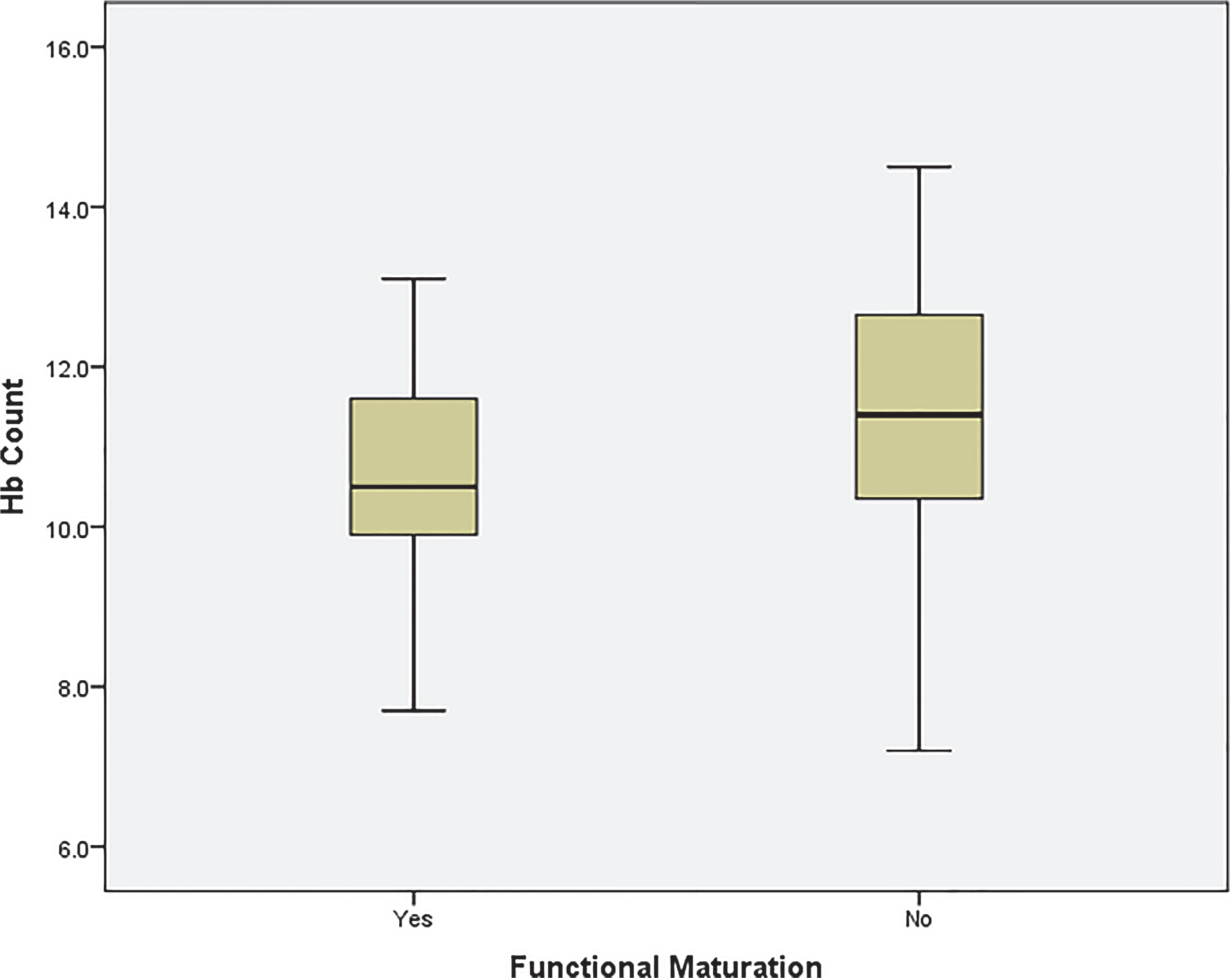
Variation in haemoglobin count between mature and non-mature AVF groups.

Independent sample t-test analysis were performed to assess the relationship between each of the above blood investigations and AVF functional maturation in our study. We found that the most statistically significant predictor of functional maturation of laboratory variables was haemoglobin (P = 0.01) [Fig. 5]; with variances in both tests proven to be equally distributed in a Levene’s test for equality of variances. Other blood investigations obtained preoperatively were not found to be independently associated with functional maturation [Table 4].

**Table 4:** Continuous variables (age and blood investigations) association with functional maturation.

	Functional Maturation	N	Mean	Std. Deviation	P value
Age at creation of fistula	Yes	49	62.37	13.869	0.926
	No	36	59.97	20.029	
Urea	Yes	49	19.122	9.5929	0.243
	No	36	16.764	8.4825	
Creatinine	Yes	49	574.00	265.916	**0.033**
	No	36	458.31	209.047	
Haemoglobin	Yes	49	10.635	1.4720	**0.010**
	No	36	11.547	1.7039	
Platelets	Yes	49	252.24	136.058	0.676
	No	36	262.97	82.417	
White cells count	Yes	49	7.6110	2.21715	0.378
	No	36	8.0219	1.95970	
Neutrophils	Yes	49	5.3404	1.93037	0.899
	No	36	5.4625	1.80142	

We performed two logistic regression analysis tests for study. The first test included all the variables in our study with a P value of < 0.1; this included gender variation, history of renal transplant prior to fistula construction, haemoglobin and a medical history of being on calcium channel blockers. The Omnibus test for model coefficients had a significant p value of < 0.001, the overall prediction accuracy of the model was 77.8% with the independent predictor for nonmaturation being a female gender (P = 0.04) and a history of calcium channel blockers (P = 0.034).

In addition to those variables, we performed a second multiple regression test and included variables that have been reported in the literature to be associated with fistula maturation, namely age, history of diabetes, smoking, hypertension, hyperlipidaemia, warfarin, congestive cardiac failure, history of starting HD prior to AVF placement, history of dialysing through CVC, and history of an attempted AVF. Omnibus test for model coefficients had a significant p value of 0.016, the overall prediction accuracy of the model was 71.7%, and as in the first model, the only independent predictor for functional nonmaturation was a female gender (P = 0.011).

## Discussion

It has been established that a functional AVF access is the least likely to be associated with thrombosis, infection, hospital admissions, secondary interventions to maintain patency and death [15,16]. However, the process of AVF maturation is complex and remains poorly understood despite numerous studies looking into the pathophysiology of the process and biomechanical factors associated with maturation. Intimal hyperplasia (IH) has been identified as the main reason behind non-maturation in the newly formed arteriovenous conduit, it is the process of cellular proliferation within the inner most layer of the vessel resulting in remodelling of both the arterial and venous ends of the new fistula [35–37]; however factors influencing this process are yet to be fully elucidated.

Our study included a total of 86 who had a combined total of 97 fistulas. Functional maturation was achieved in 52/97 fistulas (53.6%), however we were unable to determine functional maturation from dialysis records in 9 patients, and as such, the observed maturation rate in our study was 57.6%. Both percentages are in agreement with maturation rates reported in other studies [6–8]. Age did not follow a normal distribution in our study when explored against both gender and functional maturation using various normality tests provided by SPSS, however a Mann-Whitney U did not suggest an association between age and functional non-maturation. The functional maturation process in our study was statistically influenced by a female gender (P = 0.004), previous history of a kidney transplant (P = 0.036), patient on a calcium channel blocker at the time of AVF formation (P = 001) and haemoglobin levels. Out of those factors, functional non-maturation was associated with female gender and increased average of haemoglobin, while successful functional maturation was associated with a previous history of renal transplant and calcium channel blockers.

Logistic regression analysis test that included all the variables in our study that had a P value of < 0.1; (female gender, history of renal transplant, haemoglobin and being on a calcium channel blocker), showed that the only independent predictors for functional nonmaturation being female gender (P = 0.04) and a history of calcium channel blockers (P = 0.034), while the overall prediction accuracy of the model was 77.8%. Another model with the addition of other variables that have been reported elsewhere in the literature to be significantly associated with fistula functional maturation was performed; the overall prediction of this second model was 71.7%.The only independent predictor for functional nonmaturation was a female gender (P = 0.011).

Although old age (> 65) was shown to be significantly associated with non-maturation in previous studies [23,24], this however is by no means a consistent finding. Indeed many other authors found no association between age and maturation, including Renaud et al who reported similar maturation rates across all age groups in their study of 280 primary AVFs. In their study only female gender and a tunnelled catheter were significantly associated with non-maturation [30]; we report similar findings with regards to age and gender, however, in our study a history of tunnelled catheters was not associated with functional non-maturation. Also, Lok et al reported a comparable five years cumulative patency using 65 years as a cut-off point (64.7% in the ≥ 65 group and 71.4% in the < 65 group). They argued that age should not be a limiting factor when deciding which patients should have which vascular access procedure [8]. Those differences can be related to a different population, or confounding factors from other factors that might have influenced the maturation process.

We found that a lower haemoglobin level was associated with better functional maturation rates; we hypothesise that this might be explained by the up-regulation of endothelial Nitric Oxide Synthase (eNOS) in the newly formed conduit, leading to increased production of Nitric Oxide (NO) and Haeme Oxygenase-1 (HO-1) as a result of the relative hypoxia status caused by lower haemoglobin levels in the blood. NO is associated with vasodilation and decreased cellular proliferation [38], whereas HO-1 has been shown to inhibit proliferation of vascular smooth muscle cells, platelet aggregation, and vasospasm [3]. However, those are mere speculations and further studies aimed to specifically test the association of those markers and fistula maturation are needed. The significant association between calcium channel blockers and successful functional maturation is possibly mediated through the vasodilation effect commonly caused by most of those therapeutic agents.

Our findings contradict some of those reported by other authors. Nonmaturation in study by Feldman et al of 348 HD patients found was associated with a history of stroke, transient ischaemic attack, increasing age and dependence on dialysis when the fistula was created [39]. However, a study by Lee et al evaluated factors affecting cumulative access survival of AVF; they reported that age, race, diabetes, gender and peripheral vascular disease did not show significant association with access survival, with the only predictor of poor outcome being the number of salvage procedures; the higher number of secondary interventions required, the less likely for the fistula to last for a long period [25]. Those findings—with the exception of gender—were mirrored in our study.

Limitations of the study was the retrospective nature of data collection, missing data from medical records, certain continuous variables like age lacked a normal distribution pattern. It is important to mention that some of those fistulas deemed non-mature according the criteria we used in our study—functional maturation—would have been patent on duplex scans and fistulograms, and certainly the variation in expertise among dialysis staff should be expected to have influenced our maturation rate.

## Conclusion

While a retrospective study will inevitably suffer from inherent weaknesses in the methodology preventing it from sufficiently answering all questions concerning the association between demographic and haematological factors with AVF maturation, this paper would serve as a guide for future studies, as well as an up-to-date review of published evidence. Arteriovenous fistula maturation is a complex process with multiple factors involved (demographic, haematological and biomechanical). A female gender has been found to be associated with functional non-maturation, while a history of kidney transplant, calcium channel blocker agents and low haemoglobin levels were all associated with successful functional maturation. Logistic regression analysis showed that the only independent predictor of functional non-maturation was a female gender. In view of the conflicting evidence in the literature, large multi-centre registry-based studies with well-defined outcomes are required; however, other biomechanical factors that influence intimal hyperplasia should be considered as playing a leading role in AVF maturation.
